# Supplementary material for: Echinacoside as a Novel Ferroptosis Inducer in Hepatocellular Carcinoma: Mechanistic Insights from TP53/SLC7A11/GPX4 Pathway Modulation
Source: Int J Mol Sci. 2025 Dec 30;27(1):411. doi: 10.3390/ijms27010411 (PMC12787297; doi:10.3390/ijms27010411)
Supplement: Supplementary file 1 [file ijms-27-00411-s001.zip › ijms-4014359-supplementary.pdf]

SUPPLEMENTARY MATERIALS

Supplementary Table S1. The sequences of the mRNA

**Gene**	**Forward（5′-3′)**	**Reverse（3′-5′)**
TP53	CAGCACATGACGGAGGTTGT	TCATCCAAATACTCCACACGC
SLC7A11	CCCAGATATGCATCGTCCTT	GCAACCATGAAGAGGCATGT
GPX4	ACGTCAAATTCGATATGTTCAGC	AAGTTCCACTTGATGGCATTTC
GAPDH	TGCACCACCAACTGCTTAGC	GGCATGGACTGTGGTCATGAG

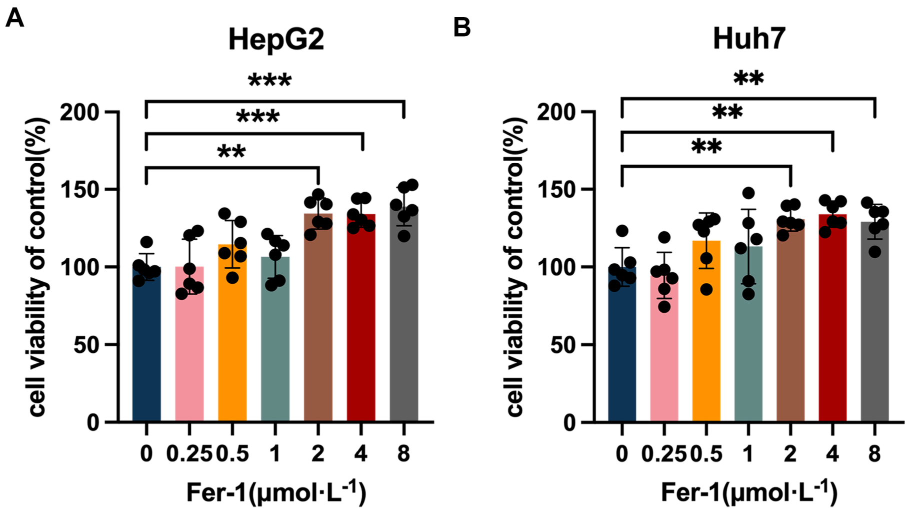

Figure S1. Effect of Fer-1 on the viability of HepG2 cells and Huh7 cells. (A) HepG2 cells were treated with different concentrations of Fer-1 for 24 hours. (B) Huh7 cells were treated with different concentrations of Fer-1 for 24 hours. Data are represented as mean ± SD (n = 3); ns = no significance, * p<0.05, ** p < 0.01 and *** p < 0.001 vs. control group. Abbreviations: Fer-1: ferrostatin-1.
